# Low Genetic Diversity and High Invasion Success of *Corbicula fluminea* (Bivalvia, Corbiculidae) (Müller, 1774) in Portugal

**DOI:** 10.1371/journal.pone.0158108

**Published:** 2016-07-08

**Authors:** Cidália Gomes, Ronaldo Sousa, Tito Mendes, Rui Borges, Pedro Vilares, Vitor Vasconcelos, Lúcia Guilhermino, Agostinho Antunes

**Affiliations:** 1 CIIMAR/CIMAR - Interdisciplinary Centre of Marine and Environmental Research, University of Porto, Terminal de Cruzeiros do Porto de Leixões, Av. General Norton de Matos, s/n, 4450–208, Porto, Portugal; 2 ICBAS - Institute of the Biomedical Sciences Abel Salazar, University of Porto, Department of Populations Study, Laboratory of Ecotoxicology, Rua de Jorge Viterbo Ferreira, 228, 4050–313, Porto, Portugal; 3 CBMA - Centre of Molecular and Environmental Biology, Department of Biology, University of Minho, Campus de Gualtar, 4710–057, Braga, Portugal; 4 FCUP - Department of Biology, Faculty of Sciences, University of Porto, Rua do Campo Alegre, s/n Edifício FC5, 4169–007, Porto, Portugal; Australian Museum, AUSTRALIA

## Abstract

The Asian clam, *Corbicula fluminea*, is an invasive alien species (IAS) originally from Asia that has spread worldwide causing major ecological and economic impacts in aquatic ecosystems. Here, we evaluated *C*. *fluminea* genetic (using COI mtDNA, CYTb mtDNA and 18S rDNA gene markers), morphometric and sperm morphology variation in Portuguese freshwater ecosystems. The COI marker revealed a single haplotype, which belongs to the Asian FW5 invasive lineage, suggesting a common origin for all the 13 Portuguese *C*. *fluminea* populations analysed. Morphometric analyses showed differences between the populations colonizing the North (with the exception of the Lima River) and the Centre/South ecosystems. The sperm morphology examination revealed the presence of biflagellate sperm, a distinctive character of the invasive androgenetic lineages. The low genetic variability of the Portuguese *C*. *fluminea* populations and the pattern of sperm morphology have been illuminating for understanding the demographic history of this invasive species. We hypothesize that these populations were derived from a unique introductory event of a *Corbicula fluminea* FW5 invasive androgenic lineage in the Tejo River, which subsequently dispersed to other Portuguese freshwater ecosystems. The *C*. *fluminea* asexual reproductive mode may have assisted these populations to become highly invasive despite the low genetic diversity.

## Introduction

Biological invasions by bivalve mollusc species have become a worldwide problem due to their dispersal capacity and effects on biological diversity, and ecosystems functions and services [1–3]. The Asian clam *Corbicula fluminea* (Müller, 1774) is nowadays globally distributed and its invasive success is responsible for major ecological and economic impacts [4–6]. The successful invasive behaviour of *C*. *fluminea* may be related to their biological traits, namely: (*i*) rapid growth, (*ii*) early sexual maturation, (*iii*) short life span, (*iv*) high fecundity, (*v*) high filtration rates, (*vi*) broad dispersal capacities (which include natural vectors), (*vii*) ability to inhabit different substrate types, (*viii*) competitive success over native species and (*ix*) interactions with human activities [4,7,8]. Major ecological impacts are related with trophic and non-trophic (engineering) mechanisms [8,9]. As a filter feeder, this species is responsible for great changes in phytoplankton and zooplankton communities [8,9]. *C*. *fluminea* populations may reach great densities and biomass being also consumed by higher trophic levels [8,9]. Regarding non-trophic mechanisms major effects are related with engineering activities, which may be responsible for great changes in water clarity, bioturbation of sediments, nutrient cycling and substrate colonization mainly due to the massive presence of shells [6,10–12].

Even though the ecological and economic impacts caused by *C*. *fluminea* are substantially documented, their taxonomic status is still unclear. Initially, morphology-based taxonomy led to an excessive number of species within the genus *Corbicula* [13]. This was mainly due to the observed high shell plasticity [14,15], which is attributed to different biotic (e.g. predation) and abiotic (e.g. water current, sediment) factors [16]. However, additional studies relying mostly on genetic analyses—alloenzymes [17–19] and mitochondrial cytochrome c oxidase subunit I DNA sequences [20,21]–proposed the existence of fewer species [14,22,23].

Interestingly, the genus *Corbicula* presents different reproductive strategies, being able to reproduce sexually [24,25] and asexually [26–30]. Previous reports also indicate that a rare form of asexual reproduction known as androgenesis occurs within the genus *Corbicula*. This phenomenon occurs after the self-fertilization process—by an oocyte and a biflagellate sperm, which is a distinctive character of androgenetic lineages of the genus *Corbicula* found in both native and invasive populations [25,28,31–35]–where the maternal nuclear DNA is completely removed, while the retained male pronucleus develops into an “all-male” zygote nucleus, thus giving rise to a progeny of paternal clones [26,27,31,32]. In addition, “egg parasitism” also known as “mitochondrial DNA capture” may occur between the crossing of two different androgenic lineages of the genus *Corbicula*, with the sperm from one lineage being able to fertilize the egg of another lineage [34,36]. The maternal nuclear DNA of the second lineage is generally mostly extruded from the egg, while the paternal nuclear genome continues to develop but the maternal mitochondrial DNA from the second lineage is captured in this process, giving rise to offspring that possess cytoplasmic-nuclear disjunction [34,37–42]. However, occasionally during this process, part or the entire maternal nuclear DNA is not completely expelled from the egg, giving rise to “nuclear genome capture” whereby the offspring inherits a hybrid genome [35,36,39,40]. Such distinctive reproductive modes seem to benefit *Corbicula* species fitness and may contribute to the invasive success of the four genus *Corbicula* invasive lineages. Three of these, namely FW1 (form B), FW4 (form Rlc) and FW5 (forms A/R) have been reported in the native (Eastern Asia) and in the non-native range (Europe and North America). The fourth, FW17 (form C/S) has been detected outside the native range but not yet in Eastern Asia [35,36,40,42].

Currently, *C*. *fluminea* presents a widespread geographic distribution and has invaded ecosystems throughout Europe, North and South America, and more recently North Africa [43–47]. Records indicate that *C*. *fluminea* was first detected outside its native range (Eastern Asia) in 1924 in Vancouver Island, British Columbia [43,45]. By the 1970s it had spread throughout North and South America [46] and reached Europe at least as early as the 1980s [44]. Britton and Morton [48] suggested that *C*. *fluminea* was firstly introduced in the North American continent as a food source for humans. However, the introductions into Europe and South America are believed to have occurred via ballast water [7]. Consequently, *C*. *fluminea* dispersed within the continents by different dispersal vectors—commercial transportation and other human activities but also by natural vectors such as birds and mammals—which promoted their rapid spread [7,45,49,50]. In Portugal, *C*. *fluminea* was first detected in the Tejo River in 1980 [44] and a few years later was reported in the Douro [51], Minho [23], Lima [52], Mondego [53] and Guadiana [54] Rivers. The history of introduction and further establishment of the species in the Tâmega, Tua, Sabor and Sado Rivers and Pateira de Fermentelos Lake are unknown.

The main objectives of this study were to assess the genetic variability and the phylogeography of *C*. *fluminea* Portuguese populations employing molecular, morphometric and morphological sperm analysis. The obtained results were compared with other available worldwide data of *Corbicula* spp. from native and invaded regions applying population genetics and phylogeographical inference methodologies.

## Material and Methods

### Ethics statement

The study did not involve any kind of endangered or protected species. No specific scientific research permits were required for the sample collection of these highly invasive invertebrates.

### Study area and sample collection

**Fig 1:**
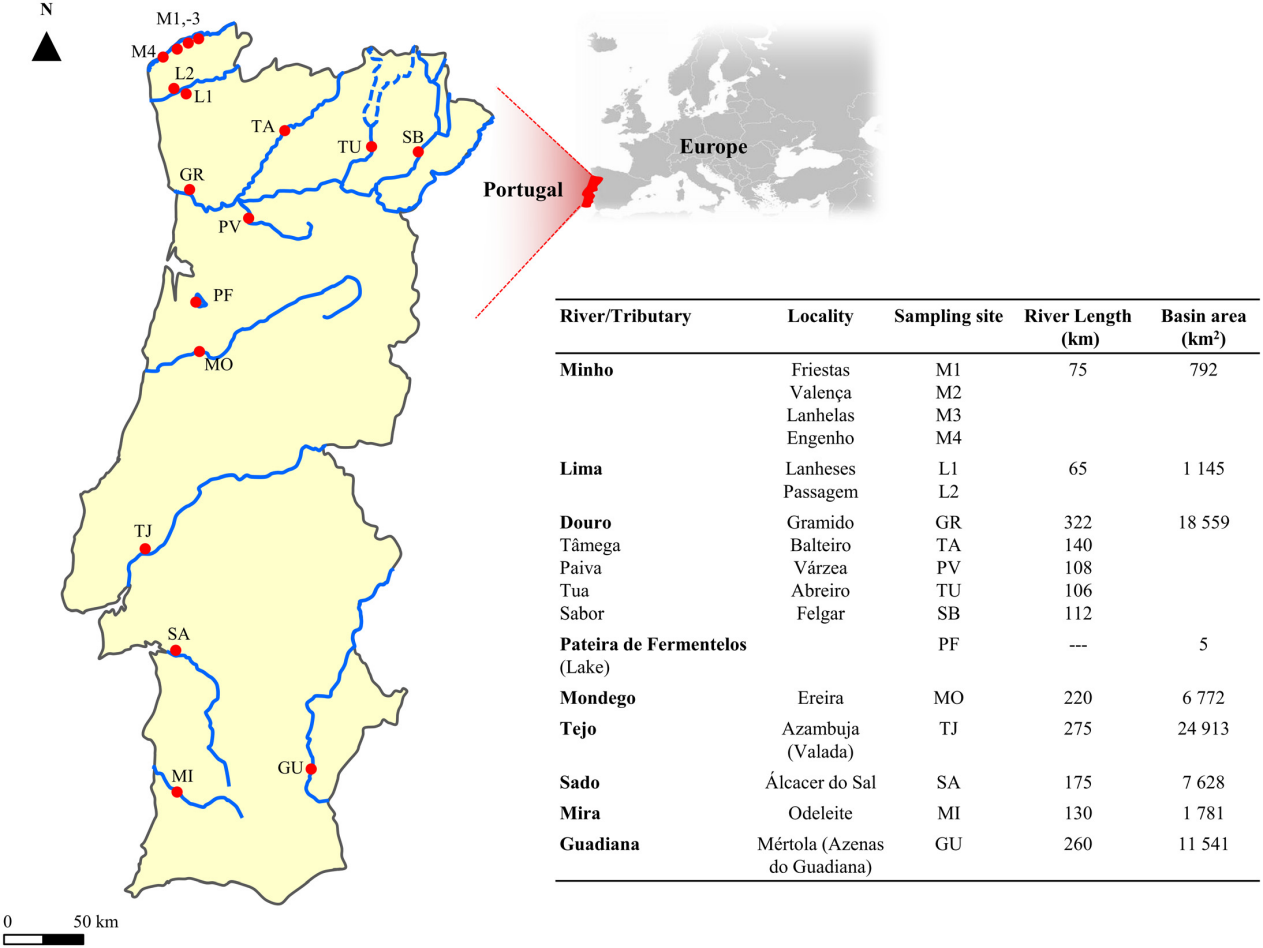
Hydrological data of the studied Portuguese rivers. Location of the sampled sites and additional information about length and area of the river and lake basins in Portuguese territory [55,56].

A total of 328 specimens of *C*. *fluminea* were randomly collected in 16 different sites belonging to 13 distinct ecosystems: Minho (four different sites, N = 100), Lima (two sites, N = 40), Tâmega (one site, N = 10), Tua (one site, N = 8), Sabor (one site, N = 10), Douro (one site, N = 10), Paiva (one site, N = 7), Mondego (one site, N = 30), Tejo (one site, N = 30), Sado (one site, N = 15), Mira (one site, N = 30), Guadiana (one site, N = 30) Rivers and Pateira de Fermentelos Lake (one site, N = 8), using a scoop net or by handpicking (Fig 1). Clams were immediately transported to the laboratory where all the soft body parts were isolated and individually stored at -80°C prior to DNA extraction.

### Genomic DNA extraction, PCR gene amplification and sequencing

Total genomic DNA was extracted from 328 samples of *C*. *fluminea* foot tissue employing the salting-out method [57]. The mitochondrial genes COI (N = 328) and CYTb (N = 110) and the nuclear gene 18S rDNA (N = 110), were amplified in a total volume of 40 μl per reaction containing: 1x PCR buffer, 2.5 mM MgCl_2_, 250 μM of each dNTP, 0.5 U of DNA Taq polymerase (Bioline, Luckenwalde, Germany), 10 pmol of a specific set of primers– LCO1490 and HC02198 for the COI [58], the HOLLAND18S1 and HOLLAND18S2 for the 18S rDNA [59] and CBF6 and CBR6 for the CYTb [60]. The following PCR cycling conditions were used for the amplification of the mtDNA COI gene: 1 min at 94°C for initial denaturation, followed by 35 cycles of 1 min at 94°C, 30 s at 45°C, 1 min at 72°C and final extension of 10 min at 72°C [58]. The 18S rDNA gene reactions were performed with the following PCR cycling parameters: 5 min at 95°C for initial denaturation, followed by 25 cycles of 4 s at 94°C, 2 min at 50°C, 1 min at 72°C and final extension of 8 min at 72°C [59]. The CYTb amplifications were performed with the following PCR cycling conditions: 2 min at 94°C for initial denaturation, followed by 35 cycles of 3 min at 94°C, 45 s at 54°C, 2 min at 72°C and final extension of 5 min at 72°C [60]. All PCR products were purified using Diffinity Rapid Tip (Diffinity Genomics, Inc, West Henrietta, NY) according to the manufacturer’s instructions. The final PCR amplifications were confirmed by electrophoresis in a 1.5% w/v agarose gel stained with ethidium bromide (Bio-Rad Laboratories Inc., California, USA) and followed by direct sequencing (Macrogen Amsterdam, Netherlands). The NCBI-BLAST program was employed for sequence identification and comparison [61,62].

### Phylogenetic analysis—mtDNA COI, CYTb and 18S rDNA genes

A total of 93 *Corbicula* spp. sequences of the mtDNA COI gene were retrieved from the NCBI-GenBank [62,63] and *Neocorbicula limosa* was used as an outgroup for phylogenetic and phylogeographic analysis. The mtDNA, 18S rDNA and CYTb sequence alignments were performed employing the default parameters of ClustalW in MEGA 6 software [64,65]. DnaSP 5.10 was used for haplotype inference [66]. Phylogenetic tree construction employed Bayesian Inference (BI) using MrBayes 3.1.2 [67] and Maximum Likelihood (ML) using PhyML 3.0.1 [68]. Both BI and ML employed the GTR + γ + I nucleotide evolutionary model based on the Akaike information criterion (with 95% confidence interval), using the jModelTest 2.1.1 [69,70]. The ML analysis used 1000 bootstrap replicates [68]. The BI analysis was performed employing 5000000 generations, the trees were sampled every 1000^th^ generation and a total of 25% of the generated trees were discarded. The tree convergence was evaluated in MrBayes by analysing the parameters set values of the Potential Scale Reduction factor (PSRF) and the Estimated sample size (ESS). In addition, further visual and numeric convergence was assessed using Tracer v1.6 software [71].

### Morphometric analysis

The shells of 275 *C*. *fluminea* specimens—from the Minho, Lima, Mondego, Sado, Mira, Tejo and Guadiana Rivers—were measured for length, height and width using a digital calliper (±0.2 mm). A Principal Component Analysis (PCA) was carried out using the three morphological measurements and the determination of the PCA components was performed using the correlation matrix in the “princomp” function of the R statistical software [72].

### Sperm morphology

A sample of *Corbicula fluminea* (N = 10 from the Douro River) was collected to perform sperm morphology analyses. The sperm was obtained by collecting one drop of the specimens fresh gonadal tissue/fluid [73] in a glass slide and optical microscopy at 100x magnification was employed to observe the spermatozoa.

## Results

### Mitochondrial DNA—COI gene

**Fig 2:**
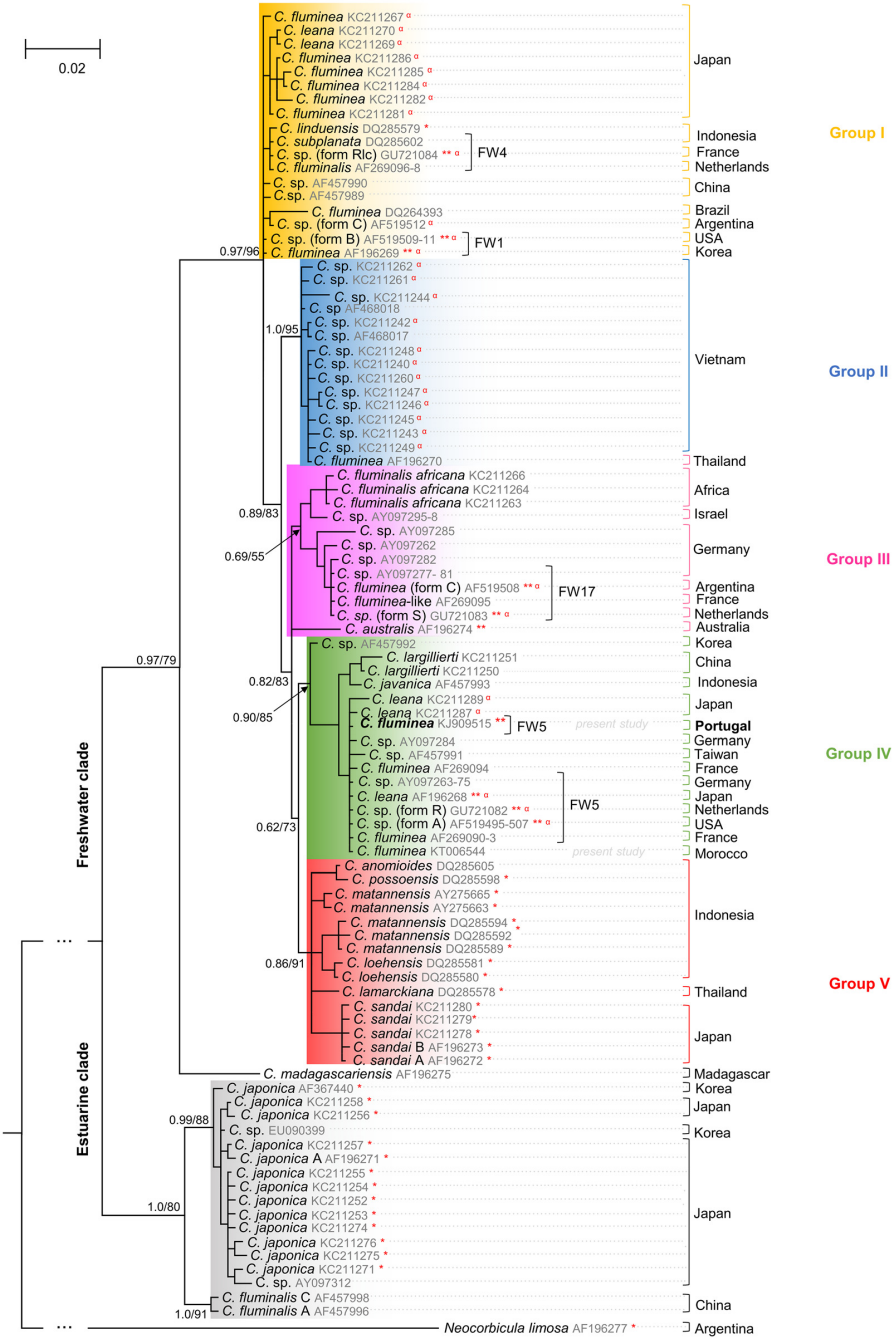
Bayesian phylogenetic tree of the mtDNA COI gene from *Corbicula* genus. Both Bayesian Inference posterior probabilities (BI) and Maximum Likelihood bootstrap values (ML) are indicated at the nodes.* Indicates the presence of monoflagellate sperm, ** indicates the presence of biflagellate sperm, ^**α**^ represents androgenetic lineages confirmed by cytological studies [25,32,73,74].

The obtained mtDNA COI sequences from all the 328 analysed individuals presented a unique haplotype which was phylogenetically compared with 93 other sequences from worldwide *Corbicula* spp. populations retrieved from GenBank [61–63]. Both BI and ML inferences implemented to reconstruct phylogenetic relationships between haplotypes displayed similar topologies (Fig 2).

The phylogenetic analysis demonstrated the existence of two well supported clades: the estuarine and the freshwater (1.0/80 and 0.97/79 support values, respectively). The latter clade splits into five groups—I, II, III, IV and V—which includes *Corbicula* specimens from different geographical ranges within Asia, Europe, North and South America, Africa and Oceania (0.97/96 node support) and *C*. *madagascariensis*, which is an outgroup of the freshwater *Corbicula* spp. lineages. For phylogenetic analysis purposes the classification “Group I-V” was employed in this study and does not imply the existence of haplotype similarity.

The evaluation of Portuguese freshwater populations—Minho, Lima, Tâmega, Tua, Sabor, Douro, Paiva, Tejo, Sado, Mira, Guadiana Rivers and Pateira de Fermentelos Lake—revealed only one mtDNA COI haplotype (belonging to group IV with 0.90/85 node support), which is identical to previously reported haplotypes from Europe (form R), North America (form A), South America and the FW5 invasive lineage from Asia (Fig 2).

Group I (0.97/96 node support) encompasses two invasive lineages, the FW1 (form B) from Asia and North America and the FW4 (form Rlc) present in Asia and Europe, whereas group IV (0.90/85 node support) represents the invasive lineage FW5 (form A/R) from Asia, Europe and North America. Both of these groups include COI haplotypes from both the native and non-native range. Groups II and V (1.0/95 and 0.86/91 node support, respectively) are strictly confined to Eastern Asia. Group III (0.69/55 node support) is the only group that includes most of the genus *Corbicula* haplotypes from the non-Asian range, namely from: Europe, South America, Africa and Oceania (with the exception of Israel which is a Western Asian country), as well as the FW17 (C/S form), detected exclusively in non-native regions, namely Europe, Africa and South America.

### CYTb and 18S rDNA genes

A subsample comprising a total of 110 specimens was used to evaluate the genetic variability of the mitochondrial CYTb and the nuclear 18S rDNA genes. The analysis revealed that only two haplotypes—one for the 18S rDNA (accession no. KT878642) and one for CTYb (accession no. KT878643)–were detected in Portuguese *C*. *fluminea* populations. This finding, suggests that Portuguese *C*. *fluminea* populations present a low genetic variability for these two markers. Further analyses employing these two markers were not carried out due to insufficient data available in the Genbank database.

### Morphometric analysis

**Fig 3:**
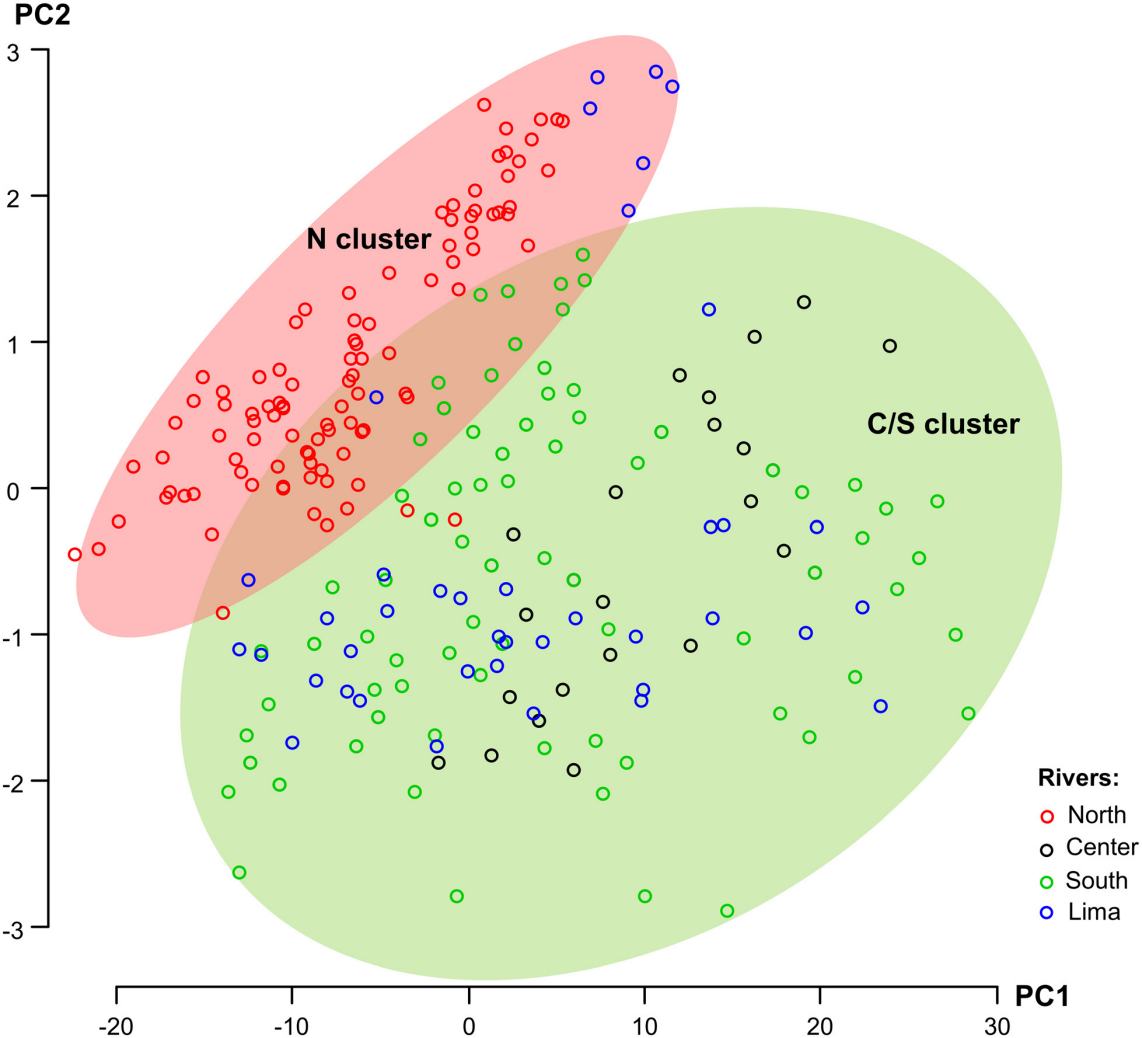
Principal Component Analysis. The PCA showing the relationship of the PC1 and PC2 components of *Corbicula fluminea* populations from rivers of the North, Centre and South of Portugal, and the Lima River (North Portugal). Each circle represents one specimen corresponding to a specific river. The N cluster in the light red oval circle represents *C*. *fluminea* populations from the northern rivers and C/S in light green oval represents the centre and southern rivers cluster.

The two PCA components explaining most variation (in total 99.4%) were jointly plotted to seek clusters related to the river/region location of each specimen (Fig 3). The PC1 and PC2 components suggest the existence of two clusters: north cluster (N), which includes *C*. *fluminea* specimens from the Minho River; and centre/south cluster (C/S) comprising specimens from Mondego, Tejo, Sado, Mira, Guadiana Rivers. However, the majority (31) of the specimens from the Lima River (N = 40), located in the North of Portugal, grouped within the C/S cluster. Therefore, morphologically they present more similarities with the *C*. *fluminea* populations from the centre and south.

### Sperm morphology

**Fig 4:**
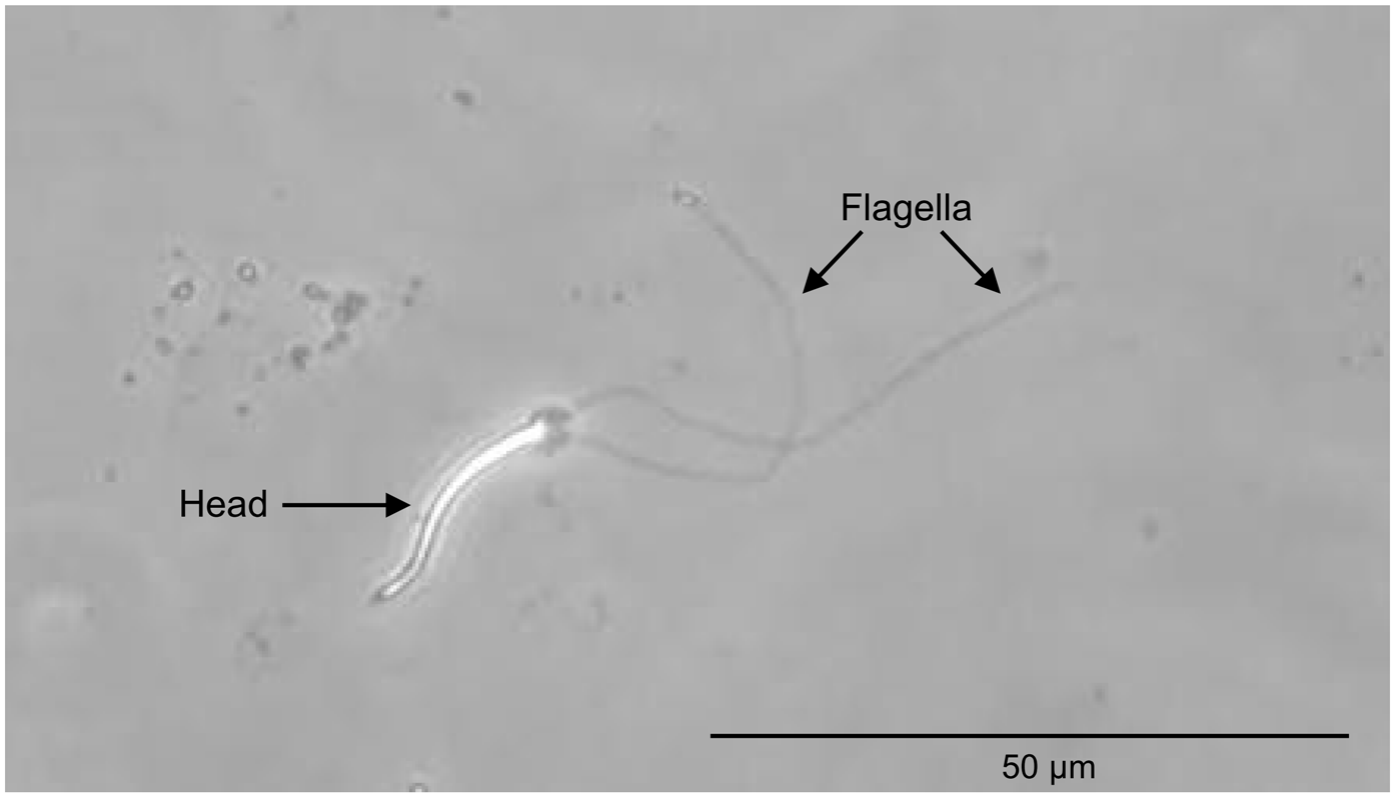
Sperm morphology of *Corbicula fluminea* from Portugal. Biflagellate sperm of a specimen of *C*. *fluminea* from the Douro River (Microscopy photograph acquired by Olympus SZX10 microscope with an integrated Olympus D72 camera).

The sperm morphology analysis of the subset *C*. *fluminea* inhabiting the Douro River clearly revealed the presence of biflagellate spermatozoa (Fig 4), a distinctive character of androgenetic lineages of the genus *Corbicula*, which are associated with high invasive potential but low genetic variability [35,40–42,74].

## Discussion

### Genetic and morphological analysis of the Portuguese *Corbicula fluminea* populations

The database sequence comparisons and the phylogenetic inference revealed the existence of a unique mtDNA COI haplotype in the studied Portuguese *C*. *fluminea* populations belonging to group IV. This haplotype is identical to the European haplotype I [20], the North American haplotype form A [21] and the Asian FW5 haplotype [33]. In addition, the FW5 haplotype comprehends the majority of *Corbicula* spp. with biflagellate sperm which is indicative this lineage reproduces through androgenesis, a rare form of asexual reproduction [40,42].

A previous study also reported the existence of this mtDNA COI haplotype in *C*. *fluminea* populations of the Minho and Lima Rivers (38 out of 41 individuals analysed in six different sampling sites) and three other rare haplotypes (each represented by only one specimen) in the Minho River [16]. However, in the present study, those rare haplotypes were not observed despite analysis of an eight times larger sample size (N = 328). The absence of the rare haplotype most probably resulted from the erosion of the genetic variability due to *C*. *fluminea* massive mortality events that occur recurrently in the Minho River [75,76] potentially leading to the loss of these rare alleles by genetic drift after a reduction in the population size [77]. Even though the *C*. *fluminea* population recovered rapidly from these die-offs and attained its previous biomass and density [75], our data shows the lack of variation of the mtDNA COI in a considerable large number of individuals from the Minho River (N = 100).

The genetic analysis of the mtDNA CYTb and 18S rDNA also yielded only one sequence for each marker in the studied *C*. *fluminea* populations. The mtDNA CYTb haplotype has also been reported in Japanese populations [60]. The 18S rDNA sequence corresponds to the same previously found in other European and North American populations namely, Spain [78], the United Kingdom [79] and the USA [80,81]. Therefore, the present study indicates a low genetic variability within the both mitochondrial (COI) and the nuclear markers (18S) in *C*. *fluminea* populations from the main Portuguese basins. Thus, this seems to be a general pattern in *C*. *fluminea* Portuguese populations. In addition, the sperm morphology revealed that at least one sample of the Portuguese *C*. *fluminea* lineage is biflagellate, a distinctive character of the asexual androgenetic lineages [28,31]. Given the lack of genetic variability detected in the studied populations with the employed genetic markers, we hypothesize that the Portuguese *C*. *fluminea* has derived from an androgenetic invasive asexual lineage with low mitochondrial genetic variability. In fact, some case-studies have reported successful invasions with low or no genetic variation in animals and plants regardless of their reproduction mode—in Africa diverse genotypes of the water flea *Daphnia pulex* have been replaced by a single non-native clone from the American continent [82], introduced populations of invasive Argentine ant *Linepithema humile* in California present a loss of genetic diversity which is associated with reduced intraspecific aggression and form interspecific dominant supercolonies [83], the Meditterranean bluespotted cornetfish *Fistularia commersonii* exhibits low genetic variability in comparison to the Indo-pacific native range [84] and the invasive water hyacinth *Eichhornia crassipes* from the Amazon basin presents one main clonal genotype in China [85,86]. In this case, *C*. *fluminea* asexual reproductive mode may assist these populations to become highly invasive despite their observed low genetic diversity [87,88]. However, further studies using other nuclear markers are required to confirm the low genetic variability at the nuclear level. Despite the low genetic variation in the mtDNA (COI and CYTb) and rDNA (18S) in *C*. *fluminea* populations found in the present study, we hypothesize that an asexual reproduction strategy might seem to increase their reproductive potential, thus contributing to their high invasive success [35,40–42,74]. Therefore, considering the invasive history of *C*. *fluminea* in Portugal, we hypothesize that the low genetic variability found in populations from main rivers all over the country, is a result of the introduction of an asexual lineage with a reduced genetic pool in Tagus River—where this species was first reported –that rapidly spread to other Portuguese freshwater ecosystems. This spread may have occurred through different dispersal mechanisms that may include human activities or natural dispersion by birds, mammals and fish as previously reported [4,7,49,89]. While the Portuguese *C*. *fluminea* populations exhibited low genetic variability, morphological differences have been detected in the present study (Fig 3). Two morphotypes were observed, one corresponding to *C*. *fluminea* populations from the northern rivers and, the other, corresponding to populations from centre/southern rivers. The exception is the population of the Lima River—located in the north of Portugal—that is morphologically more similar to Centre/South populations than to other northern populations. The observed morphological differences may be attributed to biotic or abiotic factors that influence shell morphology that may include avoidance of predation and parasitism, different current flow conditions, type of substratum, conductivity and calcium availability, among other factors [90–94]. The morphometric analysis of the studied *C*. *fluminea* populations may provide some ecologic insight, but further studies employing a hierarchal experimental design—composed of a robust *C*. *fluminea* sampling, evaluation of densities and the evaluation of both biotic and abiotic factors in these ecosystems—would be necessary to acquire a deeper ecological knowledge of the spatial variability of this species.

### Global haplotype diversity and distribution of the genus *Corbicula*

**Fig 5:**
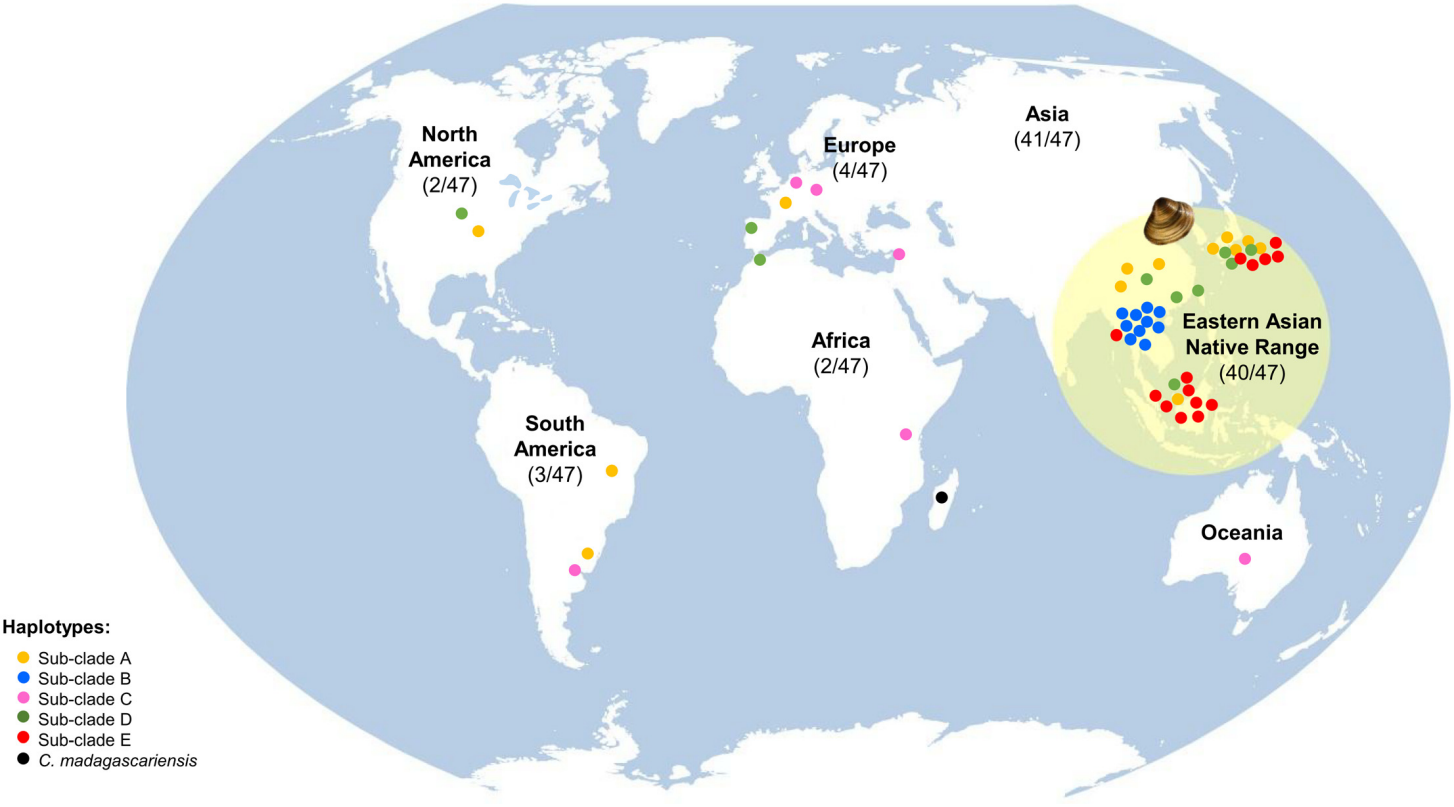
Worldwide map representing the *Corbicula* spp. distribution from the native and the non-native range. N indicates the number of haplotypes. Exclusively from the native-range two groups II and V (N = 10 haplotypes and N = 14 haplotypes, respectively). Group I is represented by 9 haplotypes and group IV by 7. The non-native range group I represents 4 haplotypes (North America, N = 1; South America, N = 2 and Europe N = 1). Group III englobes a total of 6 haplotypes; (Africa, N = 1; South America, N = 1; Europe N = 3 and Oceania, N = 1). Group IV presents a total of 3 haplotypes (North America N = 1, Europe N = 1 and Africa N = 1).

Most of the groups resolved in the phylogenetic inference present polytomies (Fig 2), indicating that *Corbicula* spp. dispersal occurred in a short temporal scale [95]. From a global perspective, we can observe that the native range presents higher *Corbicula* spp. haplotype diversity– 40 out of 47 haplotypes—in comparison to the invaded regions (Fig 5). However, the confinement or the absence from the species’ native range of some *Corbicula* spp. (groups II, V and III, respectively) still remains to be explained [74]. Perhaps physiological and environmental constraints and/or *Corbicula* spp. habitats that are less subjected to human mediated activities may in fact be inhibiting the spread of these haplotypes that derive from well-established populations [4]. Further studies should be performed to address these questions.

### The dispersal trajectory of the *Corbicula* spp. invasive lineages

**Fig 6:**
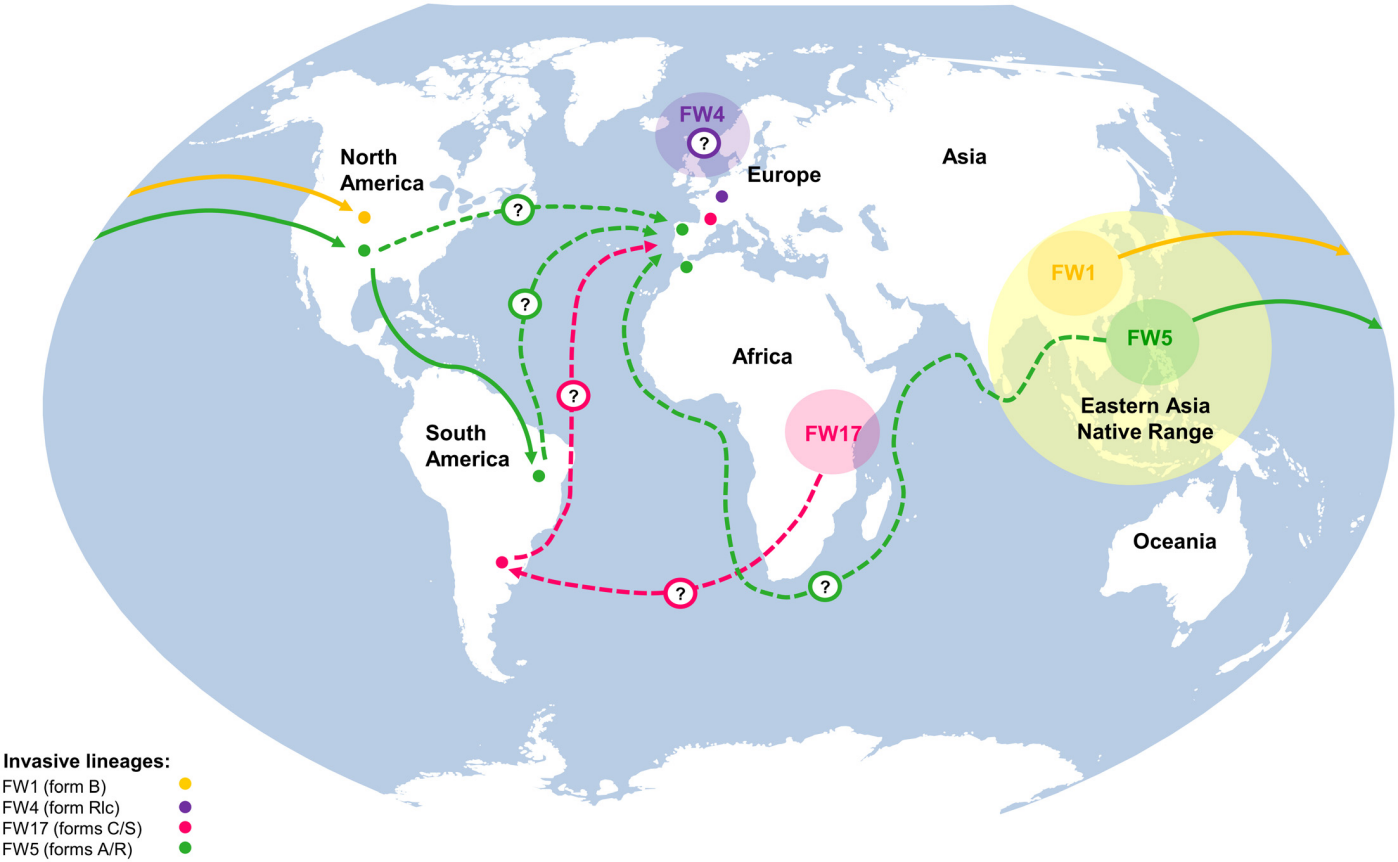
Dispersal routes of *Corbicula* spp. invasive lineages. Established *Corbicula* spp. dispersal routes are represented by continuous lines and dash lines correspond to other possible spread routes.

It is generally accepted that the *Corbicula* spp. invasion in Europe was exclusively by water ballast transport from America [96]. However, recent genetic studies are not able to confirm whether the primary introduction of the invasive lineage FW5 (form A/R) in Europe was via North and/or South America [74]. However, we cannot exclude the hypothesis that the *Corbicula* spp. may have also been introduced into the European continent directly from the Asian populations (Fig 6). The FW1 (form B) and the FW4 (form Rlc) invasive lineages (Figs 4 and 6) both clustered in group I. It has been proposed that both lineages may possess the same mitochondrial ancestor due to the detection of only one nucleotide difference in the mtDNA COI gene [74]. Interestingly, the FW17 (form C/S) invasive lineage has not been detected in the eastern native range *Corbicula* sp. (Figs 4 and 5). In fact, a recent population genetic study [74] corroborates this result and hypothesizes an introduction of the FW17 invasive lineage from the African continent and subsequent spread to the South America and subsequently into Europe. Nevertheless, inferring introductions routes for the *Corbicula* species is indeed an arduous task, especially when considering the existent taxonomic controversy in this genus. Perhaps an integrative approach employing ecology, morphology and genetic techniques may provide further insights regarding the invasive dispersal trajectory of this IAS.

## Conclusions

*C*. *fluminea* populations from the main Rivers in Portugal revealed a low genetic variability with the employed genetic markers—COI, CYTb and 18S –despite the large number of individuals detected in the studied ecosystems. The mtDNA COI and the presence of biflagellate sperm indicate that Portuguese *C*. *fluminea* populations belong to the FW5 androgenetic invasive lineage. Thus, we suggest that a reduced genetic pool was probably recently introduced first in the Tagus River and afterwards spread quickly to other Portuguese freshwater ecosystems. At the moment is not possible to unambiguously infer neither the *C*. *fluminea* primary introductory route(s) within Portugal nor the main population source (North America and/or South America or directly from Asia).
